# Conversion of a UO_2_^2+^ Precursor to UH^+^ and U^+^ Using Tandem Mass Spectrometry to Remove Both “yl” Oxo Ligands

**DOI:** 10.1021/jasms.3c00260

**Published:** 2023-10-16

**Authors:** Justin G. Terhorst, Theodore A. Corcovilos, Michael J. van Stipdonk

**Affiliations:** † Department of Chemistry, Duquesne University, 600 Forbes Avenue, Pittsburgh, Pennsylvania 15282, United States; ‡ Department of Physics, Duquesne University, 600 Forbes Avenue, Pittsburgh, Pennsylvania 15282, United States

## Abstract

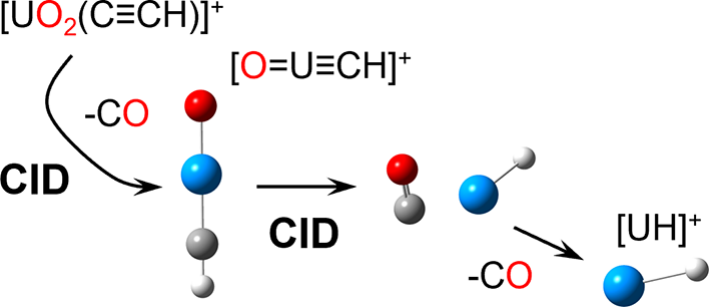

Multiple-stage collision-induced dissociation (CID) of a uranyl propiolate cation, [UO_2_(O_2_C–C≡CH)]^+^, can be used to prepare the U-methylidyne species [O=U≡CH]^+^ [*J. Am. Soc. Mass Spectrom.***2019**, *30*, 796–805]. Here, we report that CID of [O=U≡CH]^+^ causes elimination of CO to create [UH]^+^, followed by a loss of H^•^ to generate U^+^. A feasible, multiple-step pathway for the generation of [UH]^+^ was identified using DFT calculations. These results provide the first demonstration that multiple-stage CID can be used to prepare both U^+^ and UH^+^ directly from a UO_2_^2+^ precursor for the subsequent investigation of ion–molecule reactivity.

## Introduction

The high stability and inertness of U=O bonds make activation and/or functionalization of UO_2_^2+^ and UO_2_^+^ challenging.^1−6^ There is ample evidence that U=O bonds can be activated and substituted using collision-induced dissociation (CID) in multidimensional (MS^*n*^) tandem MS experiments.^7−14^ Recently, we showed that CID of the organometallic species [UO_2_(C≡CH)]^+^ induces the elimination of CO to furnish [OUCH]^+^.^12^ Density functional theory (DFT) calculations strongly supported the conclusion that the [OUCH]^+^ ion is an oxouranium-methylidyne ([O=U≡CH]^+^) product.

In general, the formation of [O=U≡CH]^+^ is important because it might be used as an intermediate to prepare new U-containing ions for the investigation of intrinsic structure, bonding, and reactivity. As we describe below, the CID of the methylidyne species causes elimination of CO to create [UH]^+^. An additional dissociation step causes the elimination of H^•^ to generate U^+^. To the best of our knowledge, this is the first demonstration that the stable UO_2_^2+^ ion can be converted to low oxidation state species such as [UH]^+^ and subsequently U^+^ for studies of intrinsic reactivity using an ion trap mass spectrometer.

## Experimental and Computational

**Figure 1:**
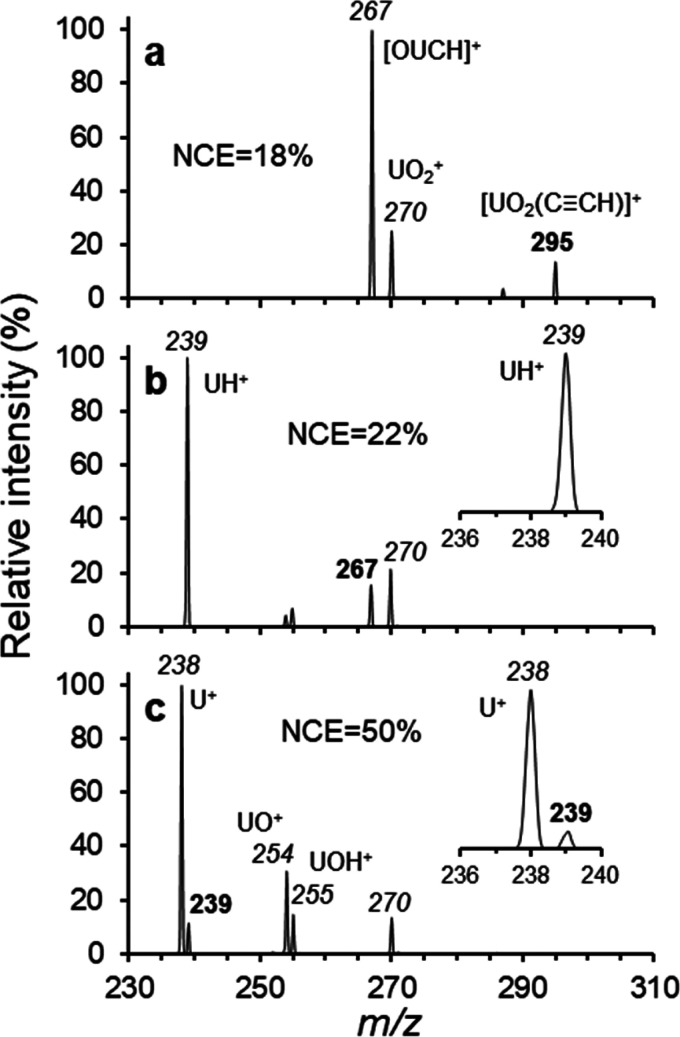
Product ion spectra generated by MS^*n*^ of [UO_2_(O_2_C≡CH)]^+^ precursor: (a) CID (MS^5^ stage) of [UO_2_C≡CH]^+^, (b) CID (MS^6^ stage) of [OUCH]^+^ and (c) CID (MS^7^ stage) of [UH]^+^. *m*/*z* values in bold represent precursor ions; those that are italicized are product ions. Normalized collision energies (NCE) used at each MS^*n*^ stage are provided in each spectrum.

Generation of [O=U≡CH]^+^ using MS^*n*^ has been described in detail elsewhere,^12^ and the experimental and computational details are provided in the Supporting Information. Briefly, [UO_2_(O_2_C–C≡CH)(CH_3_OH)_2_]^+^ was created by electrospray ionization (ESI), and the CH_3_OH ligands were eliminated in sequential CID steps (MS^2^ and MS^3^). Decarboxylation of [UO_2_(O_2_C–C≡CH)]^+^ to create [UO_2_(C≡CH)]^+^ (MS^4^) was followed by generation of [O=U≡CH]^+^ by elimination of CO (MS^5^, Figure 1a).

## Results

The product ion spectrum generated by collisional activation of [O=U≡CH]^+^ (*m*/*z* 267, MS^5^ stage) is shown in Figure 1b. The base peak appeared at *m*/*z* = 239, corresponding to the elimination of 28 Da from [O=U≡CH]^+^. Because the composition of the ion at *m*/*z* 267 was confirmed as [OUCH]^+^ by high-resolution mass measurement,^12^ a loss of 28 Da is logically attributed to the elimination of CO to yield [UH]^+^ at *m*/*z* 239. Subsequent CID of [UH]^+^ at *m*/*z* 239 (MS^6^ stage) causes the elimination of 1 Da (H^•^) to generate U^+^ at *m*/*z* 238 (Figure 1c). When including the loss of CO observed during production of [OUCH]^+^ from [UO_2_(C≡CH)]^+^, the results shown in Figure 1a demonstrate that both “yl” oxo ligands of UO_2_^2+^ can be eliminated in a multiple-stage CID experiment.

**Figure 2:**
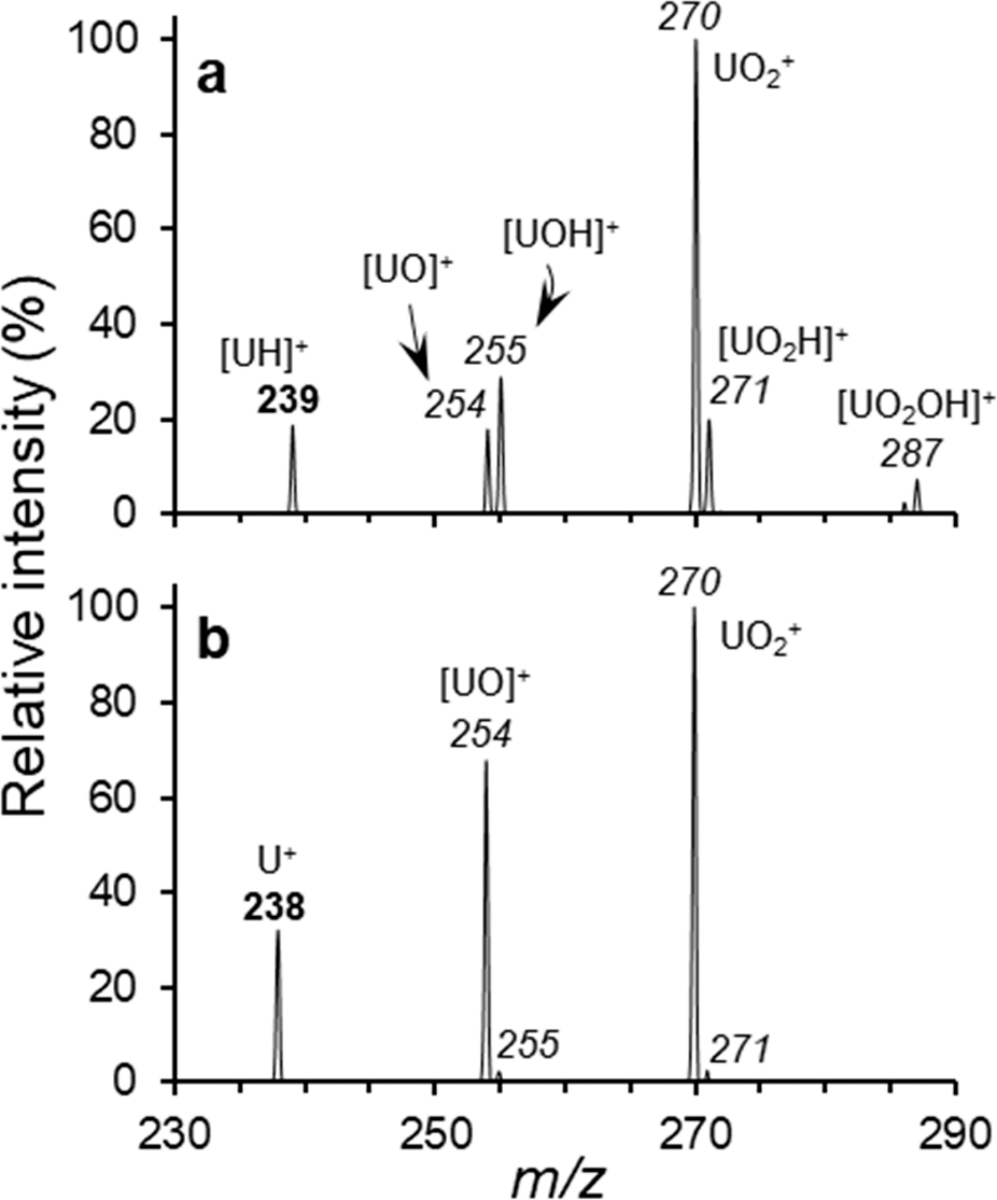
Product ion spectra generated by the isolation and storage of (a) [UH]^+^ and (b) U^+^, without imposed collisional activation, in the ion trap for 100 ms. *m*/*z* values in bold represent precursor ions; those that are italicized are product ions.

Also observed in the CID spectrum of [O=U≡CH]^+^ (Figure 1b) were peaks at *m*/*z* 254 and 255, which are assigned as [UO]^+^ and [UOH]^+^, respectively, likely created by ion–molecule reactions of [UH]^+^ and/or U^+^ with background H_2_O and O_2_ present in the ion trap during CID. To test this hypothesis, the ions at *m*/*z* 239 and 238 were independently isolated, held in the ion trap for 100 ms, and allowed to react with adventitious neutrals (typically H_2_O and O_2_). As shown in Figure 2a, isolation of [UH]^+^ at *m*/*z* 239 leads to the formation of peaks at *m*/*z* 254, 255, 270, 271, and 287. Suggested reaction pathways are summarized in Scheme S1. Formation of the product ion at *m*/*z* 271 is attributed to the reaction of [UOH]^+^ with O_2_ to generate [UO_2_H]^+^, while a subsequent reaction with adventitious H_2_O would lead to the formation of [UO_2_OH]^+^ and (neutral) H_2_. Isolation instead of U^+^ at *m*/*z* 238 for 100 ms (Figure 2b) yielded product ions at *m*/*z* 254 ([UO]^+^) and 270 (UO_2_^+^) and proposed pathways are summarized in Scheme S2. The tendency to react with O_2_ was probed using ^18^O_2_ labeled gas, which was deliberately introduced into the ion trap. We note that the product ions reported here for U^+^ are consistent with earlier studies.^15,16^ To the best of our knowledge, the intrinsic reactions of [UH]^+^ have yet to be studied and will be investigated by our laboratory in the future.

**Figure 3:**
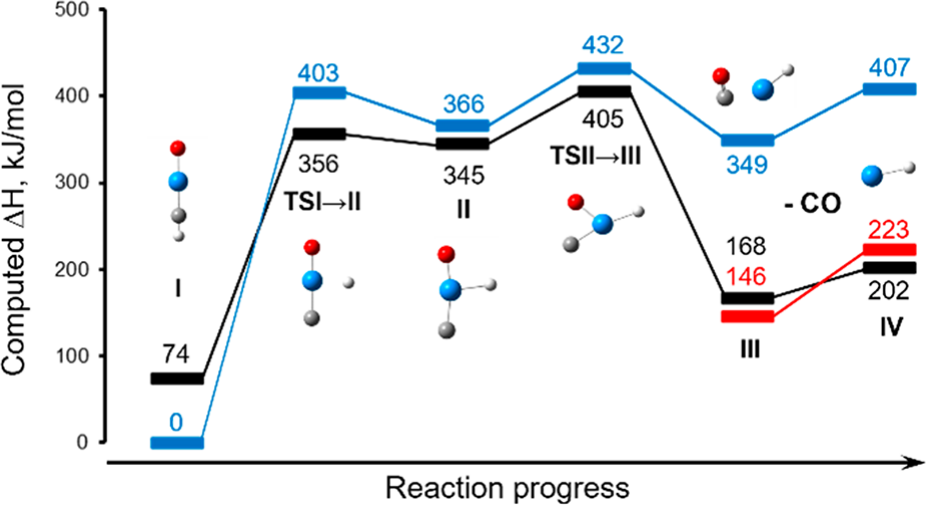
Reaction energy diagram (298 K) for the generation of [UH]^+^ from [OUCH]^+^. Relative energies for species in a singlet spin state indicated by blue symbols and lines; black symbols and lines indicate species in the triplet spin state, and red indicates the quintet states of the final two species.

Reaction energy profiles (thermally corrected enthalpies of singlet-, triplet-, and quintet-state species) generated at the B3LYP level of theory (computational methodology is provided in the SI) for dissociation of [O=U≡CH]^+^ are shown in Figure 3 (PBE0 data are provided in the Supporting Information). Calculations suggest that the formation of [UH]^+^ proceeds through a multiple-step mechanism initiated from [O=U≡CH]^+^ in the singlet spin state. The computed energy of the [O=U≡CH]^+^ precursor (**I**) in the singlet state was lower than that in the triplet state by ca. 74 kJ/mol. A crossing to the triplet surface occurs prior to the first transition state (**TSI** → **II**) to create an [O-UH-C]^+^ insertion intermediate (**II**). Rearrangement continues through **TSII** → **III**, to create a second intermediate, [UH(CO)]^+^ (**III**). Structures for **I**, **TSI** → **II**, **II**, and **TSII** → **III** failed to converge in the quintet spin state. However, **III** and [UH]^+^ (**IV**) have similar energies in the triplet and quintet states, and separation of **III** into [UH]^+^ (**IV**) was computed to require 34 and 77 kJ/mol on the triplet and quintet surfaces, respectively. An important benchmark for our study is the report by Armentrout and co-workers that the ground state of [UH]^+^, created using reaction of U^+^ with H_2_, is the quintet,^17^ with a U–H bond dissociation energy of 252.199 kJ/mol (without spin–orbit correction) compared to an experimental value of 239.270 (±5.789) kJ/mol. As a benchmark for our computational work, independent calculation of the U–H (quintet state) BDE in this study yielded a value of 254.369 kJ/mol, which is in reasonable agreement with the value of Armentrout and co-workers.^17^

The importance of “two-state reactivity” in gas-phase organometallic chemistry^18^ and the chemistry of electronic excited states^19^ has been summarized elsewhere, and the crossings of energy profiles for different spin states are consistent with our earlier studies with gas-phase U species, including^7^ the formation of [NUO]^+^ from [UO_2_(C≡N)]^+^ and generation of [O=U≡CH]^+^ from the CID of [UO_2_(C≡CH)]^+^.^14^ While spin–orbit effects were not included in our computed reaction energetics, a previous experimental and computational study of the U–H bond dissociation energy^17^ have shown that spin–orbit corrections can lower the energies of [UH]^+^ and U^+^ by 0.779 eV (75.2 kJ/mol) and 0.852 eV (85.2 kJ/mol), respectively. Based on previous reports,^20−25^ we expect that spin–orbit effects for the U 5f orbitals could enhance the coupling between the singlet, triplet, and quintet potential energy surfaces.^26^

## Conclusion

To summarize, CID of an [O=U≡CH]^+^ intermediate causes elimination of CO to create [UH]^+^ and H^•^ to form U^+^. This remarkable outcome further illustrates the advantage of using MS^*n*^ to remove both “yl” oxo ligands of the uranyl ion. DFT calculations provide a reasonable, multiple-step pathway with crossing from the singlet to triplet surface to explain the formation of the uranium hydride cation. To date, U^+^ has been formed using discharge or laser ablation sources to great effect^15,16,27,28^ and UH^+^ by the reaction of U^+^ with H_2_ or hydrocarbons.^17,29,30^ Our results show that the MS^*n*^ experiment effectively converts the stable uranyl dication to [UH]^+^ and U^+^ for investigation of ion–molecule reactivity using ion trap mass MS, thus broadening the range of species that can be created and accessed using conventional instruments equipped with ESI sources. Reliable formation of [UH]^+^ is important given the interest in actinide hydrides in catalysis and the need to understand the behavior of uranium hydrides in the context of safe storage of spent nuclear fuels.

## Supporting Information Available

Reaction energy diagram (PBE0 level of theory) for the generation of [UH]^+^ from [OUCH]^+^; proposed pathways for the reaction of [UH]^+^ and U^+^ with (neutral) O_2_ and H_2_O; experimental and computational methods; Cartesian coordinates for precursor, intermediate, and transition state structures; electronic energies and thermally corrected enthalpies for all species (PDF)
