# Supplementary material for: Conversion of a UO22+ Precursor to UH+ and U+ Using Tandem Mass Spectrometry to Remove Both “yl” Oxo Ligands
Source: J Am Soc Mass Spectrom. 2023 Oct 16;34(11):2439–42. doi: 10.1021/jasms.3c00260 (PMC10623558; doi:10.1021/jasms.3c00260)
Supplement: Supplementary file 1 — js3c00260_si_001.pdf [file js3c00260_si_001.pdf]

SUPPORTING INFORMATION

Conversion of a UO_2^{2+} Precursor to UH^+ and U^+ Using Tandem Mass Spectrometry to Remove both "yl" Oxo Ligands

Justin G. Terhorst[1], Theodore A. Corcovilos[2] and Michael J. Van Stipdonk[1*]

[1]Department of Chemistry, Duquesne University, 600 Forbes Avenue, Pittsburgh PA 15282

[2]Department of Physics, Duquesne University, 600 Forbes Avenue, Pittsburgh PA 15282

Corresponding author: Michael J. van Stipdonk (vanstipdonkm@duq.edu)

Figure S1. Reaction energy diagram (PBE0 level of theory) for generation of $[UH]^+$ from $[OUCH]^+$. Relative energies for species in singlet spin state indicated by black symbols and lines, grey symbols and lines indicated species in the triplet spin state.

Scheme S1. Proposed pathways for reaction of $[UH]^+$ with (neutral) O_2 and H_2O.

Scheme S2. Proposed pathways for reaction of U^+ with (neutral) O_2 and H_2O.

Section 1. Experimental and Computational Methodology

Section 2. Cartesian coordinates for minima and transition state structures for decomposition of $[OUCH]^+$ to create $[UH]^+$.

Section 3. Electronic energies and thermally corrected enthalpies for all species

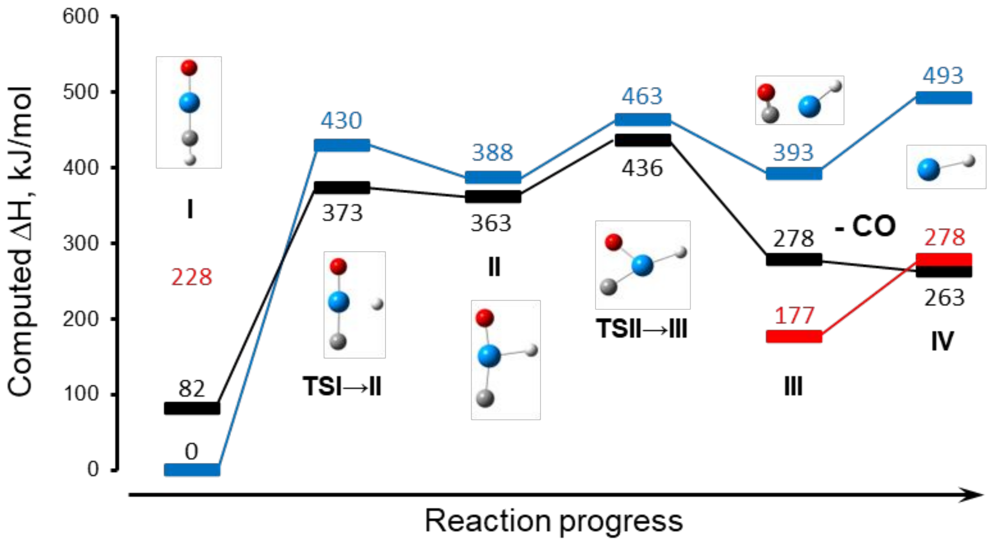
600
500
400
300
200
100
0
Computed ΔH, kJ/mol
I
228
82
0
430
373
TSI→II
388
363
II
463
436
TSII→III
393
278
- CO
177
III
493
278
263
IV
Reaction progress

Figure S1.

Scheme S1.

$$[UH]^+ + O_2 \rightarrow [UO]^+ + \bullet OH$$
m/z 239 *m/z* 254

$$[UH]^+ + O_2 \rightarrow UO_2^+ + \bullet H$$
m/z 270

$$[UH]^+ + O_2 \rightarrow [UO_2H]^+$$
m/z 271

$$[UH]^+ + H_2O \rightarrow [UOH]^+ + H_2$$
m/z 255

$$[UOH]^+ + H_2O \rightarrow [UO_2H]^+ + H_2$$
m/z 255 *m/z* 271

$$[UO_2H]^+ + H_2O \rightarrow [UO_2OH]^+ + H_2$$
m/z 271 *m/z* 287

Scheme S2.

$$U^+ + O_2 \rightarrow [UO]^+ + \bullet O$$
m/z 238 *m/z* 254

$$[UO]^+ + O_2 \rightarrow [UO_2]^+ + \bullet O$$
m/z 254 *m/z* 270

$$U^+ + H_2O \rightarrow [UO]^+ + H_2$$
m/z 238 *m/z* 254

$$[UO]^+ + H_2O \rightarrow [UO_2]^+ + H_2$$
m/z 254 *m/z* 270

Section 1.

EXPERIMENTAL METHODS

Mass Spectrometry Experiments

Methanol (CH_3OH), propiolic acid and ^{18}O-labeled O_2 were purchased from Sigma-Aldrich Chemical (St. Louis, MO) and used as received. A sample of uranyl propiolate was prepared in house by digestion of solid UO_3 (Strem Chemicals, Newburyport MA) with aqueous propiolic acid. *Caution: uranium oxide is radioactive (α- and γ-emitter), and proper shielding, waste disposal, and personal protective gear should be used when handling the material.*

Generation of the $[O{=}U{\equiv}CH]^+$ intermediate by $PTMS^n$ of uranyl propioloate precursors was performed using a previously established procedure [1]. Briefly, electrospray ionization (ESI) and CID experiments were performed on a ThermoScientific (San Jose, CA) LTQ-XL linear ion trap mass spectrometer that has been modified to allow study of ion-molecule reactions [2-4]. The atmospheric pressure ionization stack settings were optimized for maximum transmission of $[UO_2(O_2C\text{-}C{\equiv}CH)(CH_3OH)_2]^+$ (*m/z* 403) to the ion trap using the auto-tune routine via the LTQ Tune program.

For CID experiments, helium was used as the bath/buffer gas. Target ions were isolated using a width of 1.0 to 1.5 *m/z* units. The exact value was determined empirically to provide maximum ion intensity while ensuring isolation of a single isotopic peak. Values for the (mass) normalized collision energy (NCE, as defined by ThermoScientific) and activation Q were chosen empirically to enhance dissociation efficiency. For these experiments, the NCE and activation Q settings for CID of $[UO_2(C{\equiv}CH)]^+$ (Figure 1a) were 20% and 0.35, respectively. For CID of $[O{=}U{\equiv}CH]^+$ (Figure 1b), the settings for NCE and activation Q were 18% and 0.33, respectively. For CID of $[UH]^+$ (Figure 1c), the NCE and activation Q settings were 22% and 0.75, respectively.

Density Functional Theory Calculations

DFT calculations were used to determine a feasible fragmentation pathway for $[O{=}U{\equiv}CH]^+$. Geometry optimizations for potential precursor, intermediate and product ion structures were performed using the B3LYP [5-7] and PBE0 [8,9] functionals. Peterson's cc-pVTZ-PP correlation-consistent triple-zeta basis set [10] was used with the ECP60MDF Stuttgart/Koeln relativistic pseudopotential [11], and the cc-pVTZ basis set was used on was used on C, O, and H. An ultrafine integration grid was employed. Vibrational frequency calculations were used to determine whether optimized structures were true minima (no imaginary frequencies) and for thermal corrected (298.150K) energies. Transition state calculations were performed by the QST2 and QST3 methods [12]. Intrinsic reaction coordinate (IRC) calculations were used to confirm that the transition states bridged the appropriate minima. Species were modelled in the singlet, triplet and quintet spin states. All calculations were performed using the Gaussian 16 group of programs [13].

LITERATURE CITED

1.van Stipdonk, M. J.; Tatosian, I. J.; Iacovino, A. C.; Bubas, A. R.; Metzler, L.; Sherman, M. C.; Somogyi, A. Gas-phase deconstruction of UO_2^{2+}: Mass spectrometry evidence for generation of $[OU^{VI}CH]^+$ by collision-induced dissociation of $[U^{VI}O_2(C{\equiv}CH)]^+$. *J. Am. Soc. Mass Spectrom.* **2013**, *30*, 796 – 805.

2. Metzler, L. J.; Farmen, C. T.; Corcovilos, T. A.; van Stipdonk, M. J. Intrinsic Chemistry of $[OUCH]^+$: Reactions with H_2O, $CH_3C{\equiv}N$ and O_2. *Phys. Chem-Chem. Phys*. **2021**, *23*, 4475 – 4479.

3. Metzler, L. J.; Farmen, C. T.; Fry, A. N.; Seibert, M. P.; Massari, K. A.; Corcovilos, T. A.; van Stipdonk, M. J. Intrinsic Reactivity of $[OUCH]^+$: Apparent Synthesis of $[OUS]^+$ by Reaction with CS_2. *Rapid Commun Mass Spectrom*. **2022**, *36*, e9260.

4. van Stipdonk, M. J.; Perez, E. H.; Metzler, L. J.; Bubas, A. R.; Corcovilos, T; Somogyi, A. Destruction and Reconstruction of UO_2 Using Gas-phase Reactions. *Phys. Chem. – Chem. Phys.* **2021**, *23*, 11844 – 11851.

5. Becke, A. D. Density-functional thermochemistry. III. The role of exact exchange. *J. Chem. Phys*. **1993**, *98*, 5648-5652.

6. Lee, C.; Yang, W.; Parr, R. G. Development of the Colle-Salvetti correlation-energy formula into a functional of the electron density. *Phys. Rev. B*. **1988**, *37*, 785-789.

7. Stephens, P. J.; Devlin, F. J.; Chabalowski, C. F.; Frisch, M. J. Ab Initio calculation of vibrational absorption and circular dichroism spectra using density functional force fields. *J. Phys. Chem*. **1994**, *98*, 11623-11627.

8. Perdew, J. P.; Ernzerhof, M.; Burke, K. Rationale for mixing exact exchange with density functional approximations. *J. Chem. Phys*. **1996**, *105***,** 9982–9985.

9. Adamo, C.; Barone, V. Toward reliable density functional methods without adjustable parameters: The PBE0 model. *J. Chem. Phys*. **1999**, *110*, 6158–6170.

10. Peterson, K. A.; Correlation consistent basis sets for actinides. I. The Th and U atoms. *J. Chem. Phys*. **2015**, *142*, 074105.

11. Dolg, M.; Cao, X. Accurate Relativistic Small-Core Pseudopotentials for Actinides. Energy Adjustment for Uranium and First Applications to Uranium Hydride. *J. Phys. Chem. A*. **2009**, *113*, 12573-12581.

12. Peng, C.; Schlegel, H. B. Combining synchronous transit and quasi-Newton methods for finding transition states. *Israel J. Chem*. **1993**, *33*, 449 – 454.

13. Gaussian 16, Revision C.01, M. J. Frisch, G. W. Trucks, H. B. Schlegel, G. E. Scuseria, M. A. Robb, J. R. Cheeseman, G. Scalmani, V. Barone, G. A. Petersson, H. Nakatsuji, X. Li, M. Caricato, A. V. Marenich, J. Bloino, B. G. Janesko, R. Gomperts, B. Mennucci, H. P. Hratchian, J. V. Ortiz, A. F. Izmaylov, J. L. Sonnenberg, D. Williams-Young, F. Ding, F. Lipparini, F. Egidi, J. Goings, B. Peng, A. Petrone, T. Henderson, D. Ranasinghe, V. G. Zakrzewski, J. Gao, N. Rega, G. Zheng, W. Liang, M. Hada, M. Ehara, K. Toyota, R. Fukuda, J. Hasegawa, M. Ishida, T. Nakajima, Y. Honda, O. Kitao, H. Nakai, T. Vreven, K. Throssell, J. A. Montgomery, Jr., J. E.

Peralta, F. Ogliaro, M. J. Bearpark, J. J. Heyd, E. N. Brothers, K. N. Kudin, V. N. Staroverov, T. A. Keith, R. Kobayashi, J. Normand, K. Raghavachari, A. P. Rendell, J. C. Burant, S. S. Iyengar, J. Tomasi, M. Cossi, J. M. Millam, M. Klene, C. Adamo, R. Cammi, J. W. Ochterski, R. L. Martin, K. Morokuma, O. Farkas, J. B. Foresman, and D. J. Fox, Gaussian, Inc., Wallingford CT. (2016)

Section 2. Cartesian coordinates and electronic energies

<u>Singlet species</u>

Structure **I** (electronic energy: -588.471664 Hartree)

1 1

U	0.00000000	0.00000000	0.00169600
O	0.00000000	0.00000000	-1.74731000
C	0.00000000	0.00000000	1.81770200
H	0.00000000	0.00000000	2.91621100

Structure **TSI→II** (electronic energy: -588.311998 Hartree)

1 1

U	-0.27612000	0.08110900	0.53434000
O	0.65346900	-0.31846100	-0.90646700
C	-1.69124200	0.69030800	1.65760100
H	1.11304200	-0.46286100	1.78783200

Structure **II** (electronic energy: -588.32771 Hartree)

1 1

U	0.02167400	0.08442800	0.00028700
O	-1.59585800	-0.60727400	-0.00009200
C	1.79320600	-0.55248400	-0.00011600
H	-0.91931900	1.76020700	-0.00007000

Structure **TSII→III** (electronic energy: -588.300816 Hartree)

1 1

U	0.06657100	0.50684600	0.08119800

O	-0.98111900	-0.91421200	-0.23503200
C	1.18215100	-1.06171800	0.08273300
H	-0.96790000	2.15396200	0.07111000

Structure **III** (electronic energy: -588.334174 Hartree)

1 1

U	-0.30114800	0.00279500	-0.02136100
O	1.85398400	0.48821900	-0.19359100
C	1.76056100	-0.66402600	0.28265800
H	-0.75715100	1.20996400	1.48492500

Structure **IV** (electronic energy: -474.954903 Hartree)

1 1

U	0.00000000	0.00000000	0.02085900
H	0.00000000	0.00000000	-1.91900300

CO (electronic energy: -113.357253 Hartree)

0 1

O	1.82040300	0.44340500	-0.15589600
C	1.73343400	-0.60083100	0.26060400

<u>Triplet species</u>

Structure **I** (electronic energy: -588.442354 Hartree)

1 3

U	0.00000000	0.00000000	0.01046300
O	0.00000000	0.00000000	1.76925200
C	0.00000000	0.00000000	-2.00191900
H	0.00000000	0.00000000	-3.10512400

Structure **TSI→II** (electronic energy: -588.329735 Hartree)

1 3

U	-0.27263200	0.14130900	0.40753000
O	0.58530100	-0.38649700	-1.02864500
C	-1.36927600	0.82691900	1.89700900
H	0.85575600	-0.59163600	1.79741100

Structure **II** (electronic energy: -588.334927)

1 3

U	-0.13503600	-0.17091700	0.00035400
O	-1.81945900	-0.65879200	0.00000800
C	1.82655500	-0.20180700	0.00009000
H	-0.57235700	1.71639400	-0.00044300

Structure **TSII→III** (electronic energy: -588.310529)

1 3

U	-0.61311600	0.45386600	0.06255400
O	-0.75655400	-1.37336000	-0.06048700
C	0.99576100	-0.79086000	-0.18687400
H	-0.32638800	2.39523300	0.18481600

Structure **III** (electronic energy: -588.404101)

1 3

U	-0.43930500	0.10146200	-0.11531600
O	1.89630700	0.38730900	-0.29759000
C	1.86569500	-0.60171500	0.41174000
H	-0.76645100	1.14989500	1.55379800

Structure **IV** (electronic energy: -475.033283)

1 3

U	-0.30450800	0.01168900	-0.01026300
H	-0.75379100	1.20107000	1.47382700

Quintet species

Structure **III** (electronic energy -588.411977)

1 5

U	-0.45672000	0.10275800	-0.09547500
O	1.91439600	0.37994700	-0.29061600
C	1.91012000	-0.62093500	0.37963800
H	-0.81155100	1.17518200	1.55908500

Structure **IV** (electronic energy: -475.025259)

1 5

U	-0.30410900	0.01063200	-0.01158200
H	-0.75419000	1.20212700	1.47514600

Section 3. Electronic Energies and Thermally Corrected Enthalpies

B3LYP/cc-pvtz-pp/cc-pvtz

B3LYP/cc-pvtz-pp/cc-pvtz	Stucture	EE	ZPE	EE+ZPE	Δ(EE+ZPE)	kJ	Hcorr	H	ΔH	kJ
Quintet										
	**III**	-588.412	0.0103	-588.402	34.7	145.1	0.01574	-588.396	34.9	146.0
	**IV**	-475.025	0.003971	-475.021			0.007277	-475.018		
	**CO**	-113.357	0.005014	-113.352			0.008319	-113.349		
				-588.374	52.4	219.0	0.015596	-588.367	53.3	223.0
Triplet										
	**I**	-588.442	0.013212	-588.429	17.5	73.0	0.018774	-588.424	17.7	74.2
	**TSI->II**	-588.33	0.008239	-588.321	85.0	355.6	0.013594	-588.316	85.2	356.3
	**II**	-588.335	0.008917	-588.326	82.2	343.8	0.014601	-588.32	82.5	345.3
	**TSII->III**	-588.311	0.008221	-588.302	97.0	406.0	0.013065	-588.297	96.9	405.3
	**III**	-588.404	0.010406	-588.394	39.7	166.1	0.015745	-588.388	39.8	166.7
	**IV**	-475.033	0.00398	-475.029			0.007287	-475.026		
	**CO**	-113.357	0.005014	-113.352			0.008319	-113.349		
				-588.382	47.3	198.0		-588.375	48.3	201.9
Singlet										
	**I**	-588.472	0.01471	-588.457	0.0	0.0	0.019821	-588.452	0.0	0.0
	**TSI->II**	-588.312	0.00844	-588.304	96.3	402.7	0.013764	-588.298	96.4	403.3
	**II**	-588.328	0.010211	-588.317	87.5	366.1	0.015413	-588.312	87.6	366.4
	**TSII->III**	-588.301	0.008869	-588.292	103.5	433.2	0.01364	-588.287	103.3	432.3
	**III**	-588.334	0.009861	-588.324	83.2	348.2	0.015393	-588.319	83.5	349.4
	**IV**	-474.955	0.003892	-474.951			0.007198	-474.948		
	**CO**	-113.357	0.005014	-113.352			0.008319	-113.349		
				-588.303	96.5	403.6		-588.297	97.4	407.5

PBE0/ cc-pvtz-pp/cc-pvtz

B3LYP/cc-pvtz-pp/cc-pvtz	Stucture	EE	ZPE	EE+ZPE	Δ(EE+ZPE)	kJ	Hcorr	H	ΔH	kJ
Quintet										
	**III**	-588.175	0.010546	-588.164	42.0	175.9	0.015943	-588.159	42.3	176.9
	**IV**	-474.911	0.00401	-474.907			0.007316	-474.903		
	**CO**	-113.226	0.005084	-113.22			0.008388	-113.217		
				-588.127	65.5	274.0		-588.121	66.5	278.2
Triplet	**I**	-588.214	0.013497	-588.201	19.3	80.7	0.018991	-588.195	19.6	81.9
	**TSI->II**	-588.098	0.008572	-588.09	89.0	372.5	0.013825	-588.084	89.2	373.1
	**II**	-588.103	0.009345	-588.094	86.3	361.2	0.014894	-588.088	86.7	362.6
	**TSII->III**	-588.074	0.00833	-588.065	104.2	436.0	0.01334	-588.06	104.2	436.0
	**III**	-588.136	0.010626	-588.126	66.3	277.3	0.015937	-588.121	66.5	278.1
	**IV**	-474.916	0.004005	-474.912			0.007311	-474.909		
	**CO**	-113.226	0.005084	-113.22			0.008388	-113.217		
				-588.133	61.9	259.1		-588.126	62.9	263.3
Singlet	**I**	-588.247	0.015121	-588.231	0.0	0.0	0.020134	-588.226	0.0	0.0
	**TSI->II**	-588.077	0.008742	-588.068	102.6	429.2	0.014018	-588.063	102.7	429.8
	**II**	-588.094	0.010623	-588.084	92.7	387.7	0.015735	-588.079	92.7	387.9
	**TSII->III**	-588.064	0.009201	-588.055	111.0	464.3	0.013891	-588.05	110.8	463.5
	**III**	-588.092	0.010044	-588.082	93.6	391.7	0.015488	-588.077	93.9	392.8
	**IV**	-474.829	0.003907	-474.825			0.007213	-474.821		
	**CO**	-113.226	0.005084	-113.22			0.008388	-113.217		
				-588.045	116.9	489.1		-588.039	117.9	493.2
